# Investigating the impact of hearing loss on attentional networks among older individuals: an event-related potential study

**DOI:** 10.1007/s11571-024-10140-x

**Published:** 2024-06-21

**Authors:** Sankalpa Madashetty, Hari Prakash Palaniswamy, Bellur Rajashekhar

**Affiliations:** Department of Speech and Hearing, Manipal College of Health Professions, Manipal Academy of Higher Education, Manipal, Karnataka 576104 India

**Keywords:** Attention networks, ERPs, Hearing loss, Executive attention, Older individuals

## Abstract

Attention is a core cognitive domain crucial in facilitating day-to-day life. Using an attention network test (ANT) along with event-related potentials (ERPs) in older individuals with hearing loss would provide excellent information about the impact of hearing loss on attentional processes. Thus, the current study aims to understand the attentional deficits and its cortical dynamics in older individuals with and without hearing loss. The study recruited 40 participants, 20 older individuals with hearing loss and 20 age and education-matched controls with normal hearing. All the participants underwent cognitive assessment using ANT with simultaneous 32-channel EEG recording. Results revealed significant impairment in executive attention and subtle alterations in alerting and orienting attention among older individuals with hearing loss compared to their normal-hearing counterparts. These findings suggest the negative impact of hearing loss on attentional networks. In addition, ANT and ERPs provide insight into the underlying neural mechanisms in specific attention network deficits associated with hearing loss.

## Introduction

Hearing loss is one of the most common non-communicable conditions affecting millions worldwide. Research has shown interference of hearing loss in an individual’s ability to attend, understand and process speech, especially during noisy environments. Which, in turn, affects a person’s quality of life and cognitive functioning. Attention is a crucial core cognitive domain that facilitates everyday routines like selective listening, communicating, driving, and other social interactions. Therefore, it is essential to understand the attentional processes in older individuals with hearing loss.

Many studies have explored attentional processes in healthy aging individuals and other clinical populations. Specifically, there have been 14 studies published to date that assessed the Attention Network Test (ANT) in older individuals. Of these, only three explored attentional networks in healthy aging individuals (Gamboz et al. 2010; Williams et al. 2016; Young-Bernier et al. 2015). These studies report impaired executive attention among older individuals compared to healthy young adults. Studies on hearing-impaired children and adults have also shown impaired altering attention (Daza & Phillips-Silver, 2013; Ma et al. 2023). Mixed results were reported for orienting and executive attention in this population. Since both hearing loss and age have independent effects on attention networks, attention network deficits might be pronounced in individuals with ARHL.

However, there is a lack of understanding of attention and its related deficits in older individuals with hearing loss, specifically in the Attention Network Test (ANT) framework. ANT is a widely used computerized task designed to simultaneously assess alerting, orienting, and executive attention networks (Fan et al. 2002). There are several tests for assessing attention, but a triple network model by Posner and Petersen (1990) is the mainstay of attention-related studies with over 3000 studies (Online ANT database). Investigation of attention networks using ANT would provide insights into the specific attentional networks that are affected due to hearing loss in the older population.

In literature, few studies specifically explore the effect of hearing loss on attentional processes, most of them using executive functions. These include tasks like Stroop (Gussekloo et al. 2005; Valentijn et al. 2005), Erikson Flanker (Bonmassar et al. 2023) and trail making test TMT (Lin et al. 2011). However, most of these studies included self-reported hearing loss or pure tone audiometry (PTA). However, most individuals with hearing loss or aging would have speech perception difficulty in adverse listening conditions like speech in noise (SPIN) (Pichora-Fuller et al. 2015). Hence, it is necessary to evaluate attention processes in older individuals with hearing loss compared to age and education-matched controls and their correlation with SPIN.

Event related potentials (ERPs) are electrophysiological responses that are time-locked to specific cognitive, motor, or sensory events. It provides a non-invasive means of investigating the neural processes that underlie cognitive functions such as attention, memory, and language. Early processes can be observed as early as 100 msec (N100) and are known to influence the later stages of cognitive processes (Herrmann and Knight 2001); (Hillyard and Anllo-Vento 1998; Luck et al. 1990). Thus, ERP alterations can be used as sensitive biomarkers to understand the stages of neural mechanisms underlying specific disturbed cognitive processes due to pathological conditions (Sokhadze et al. 2017). So, using ERP during ANT could validate the behavioural manifestation of attention and serve as a useful biomarker.

With this, the current study aims to explore how ARHL affects attention in the framework of ANT. The specific research questions are: How does ARHL influence different attentional networks? Does attention deficit explain speech in noise perception in individuals with ARHL? Finally, to explore the electrophysiological correlates of attentional networks in individuals with ARHL.

## Method

### Participants

As a part of this study, 352 older individuals were screened for potential participants. The inclusion criteria were a score ≥ 4 in Mini-Cog and less than or equal to 25 on the Quick Neurological Screening Test (QNST) to recruit cognitively and neurologically normal individuals for the study. Individuals with a history or self-reported psychological disorders, uncorrected visual impairment, and any other causes of hearing loss other than age were excluded from the study. These individuals were assessed for their hearing thresholds using standard pure-tone audiometry and SIN-K (speech in noise test - Kannada) (Avinash et al. 2010).

**Fig. 1:**
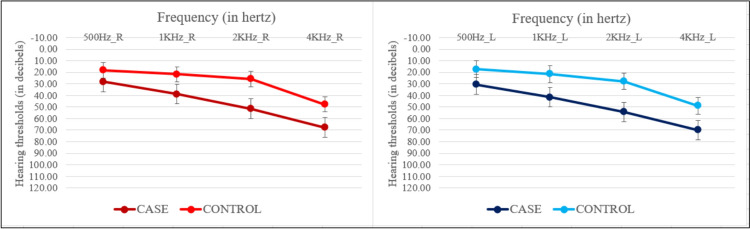
Mean pure tone thresholds for the right (red) and left (blue) ears of hearing loss and normal hearing group

Individuals with mild to moderate hearing loss were grouped into the hearing loss group (*n* = 20), and age, and education-matched individuals with less than minimal hearing loss were grouped into the normal hearing group (*n* = 20). All the participants gave written consent before the test procedure. Table 1 below shows the demographic and participant characteristics of the participants in both groups. Figure 1 represents the mean pure tone thresholds of both the groups with 1SD.

**Table 1:** Demographic information of all the participants

	Hearing loss (*n* = 20)	Normal hearing (*n* = 20)	t	*p*-value
Age (in years)	65.61 ± 5.007	64.80 ± 4.663	0.545	0.589
Gender (male/female)	11/09	14/06	–	–
Handedness (right/left/mixed)	20/0/0	20/0/0	–	–
Education (in years)	11.70 ± 2.285	12 ± 2.340	− 0.431	0.669
MiniCog scores	4.13 ± 0.458	4.25 ± 0.444	− 0.866	0.391
Right ear—Pure tone average	41.32 ± 6.51	21.92 ± 1.97	15.305	< 0.001
Left ear—Pure tone average	42.10 ± 5.8	22.16 ± 2.24	6.212	< 0.001
SNR Loss	8.283 ± 1.90	0.250 ± 2.53	11.846	< 0.001

### Test Procedure

The detailed assessment encompassed cognitive tests with simultaneous EEG Recording. The testing was done in a dimly lit, sound-treated room with comfortable participant seating.

#### Cognitive assessment

This study used well known Attention Network Test (ANT) in Eprime 3, and the paradigm used is the same as initially described by Fan et al. (2002). The experiment comprised 3 blocks with 96 trials each, totaling 288 trials. The trials were presented randomly (4 cue conditions x 2 stimulus positions x 2 stimulus directions x 3 congruency conditions x 2 repetitions). Each trial had five components: Initial fixation with random variable duration (ranging from 400 to 1600ms), then cue appeared for 100ms, followed by a middle fixation time of 400 ms. Then, the stimulus consisting of target and flankers was presented until the participant responded or for a maximum of 1700ms. Once the participant responds, the stimulus disappears, and there will be a post-stimulus fixation period of variable duration (3500 msec−first fixation duration—participant’s reaction time). Please see Fan et al. (2002) for the detailed paradigm.

The experiment involved displaying a fixation cross at the center of the screen, with cue stimuli appearing above or below the fixation cross (spatial cue), in the center (central cue), or not at all (no cue). The target stimulus consisted of five horizontal arrows or lines above or below the fixation cross. Each arrow or line was 0.55° of visual angle in length, and the distance between adjacent arrows or lines was 0.06° visual angle. The stimuli, which included a central arrow and four flankers, totaled 3.08° visual angle. These stimuli were presented either 1.06° above or below the fixation point to introduce the attentional-orienting component. The target location was always uncertain except when a spatial cue was presented.

**Fig. 2:**
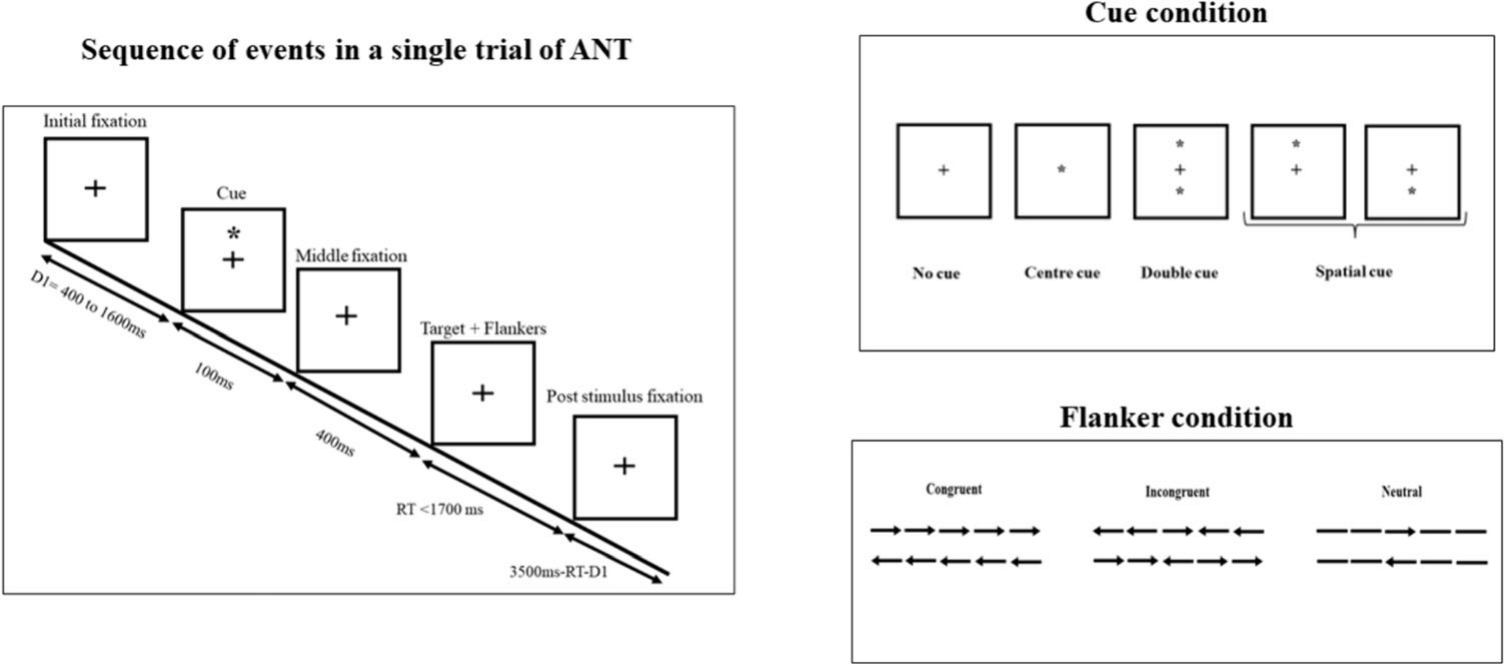
Block diagram of the ANT paradigm *Note*: The duration of each component in the trial is mentioned in milliseconds in the above figure

During the test, participants were seated 65 cm from the computer screen to maintain a consistent visual angle of the stimulus. They were instructed to focus on the fixation point at the center throughout the test. All participants were asked to quickly respond to the direction of the center arrow (target) while ignoring the surrounding arrows (flankers) using the response device (Chronos). Initially, the participants completed a practice block of 24 trials, and accuracy and reaction time feedback was provided before the experimental blocks. Figure 2 demonstrates the block diagram of the ANT paradigm, and Table 2 describes the cue condition.

The Attention Network Test is a tool designed to assess the efficiency of three distinct attention networks in the brain: alerting, orienting, and executive control. Each network plays a critical role in processing information and guiding behavior. The alerting network is responsible for achieving and maintaining a state of heightened alertness. It was measured by presenting cues that signal the appearance of a target stimulus. The difference in reaction time between trials with and without a cue assesses the efficiency of the alerting network. A faster response to cued trials indicates a more efficient alerting network.

The orienting network selects sensory input information, directing attention to specific locations or modalities. This was tested by presenting cues that indicate the location where the target will appear (above or below the fixation). The difference in reaction times between correctly cued and centrally cued or uncued trials measures the orienting network’s efficiency. An efficient orienting network is indicated by quicker responses to correctly cued trials.

The executive control network manages complex cognitive processes, including resolving conflict among responses. This was assessed through trials that presented conflicting information, such as incongruent flankers surrounding the target. The difference in reaction time between congruent (non-conflicting) and incongruent (conflicting) trials was used to gauge the efficiency of the executive control network. A smaller difference suggests a more efficient executive control network capable of swiftly resolving conflicts.

**Table 2:** Description of Cue conditions

Cue conditions	Description	
No cue	Just fixation for 100ms	Without any cue
Center cue	An asterisk (*) presented for 100ms at the location of fixation	Alerting
Double cue	Two asterisks were cueing about two possible stimuli locations- Up and Down.	Alerting with a large attentional field
Spatial cue	Asterisk is presented precisely at the stimuli position for 100ms	Valid cue; involves both alerting and orienting

#### Electroencephalography recording

A task-related EEG was recorded using an active channel amplifier with 32 electrodes placed based on a 10–20 system (ActiChamp, Brain Vision v005 10/2017). A continuous EEG with Cz as a reference was recorded with an online filter from 1 to 100 Hz and a sampling frequency of 500 Hz. Every participant actively performed the ANT experiment during the EEG Recording. Electrode impedance was maintained below 50 kΩ throughout the testing.

#### EEG data analysis

**Fig. 3:**
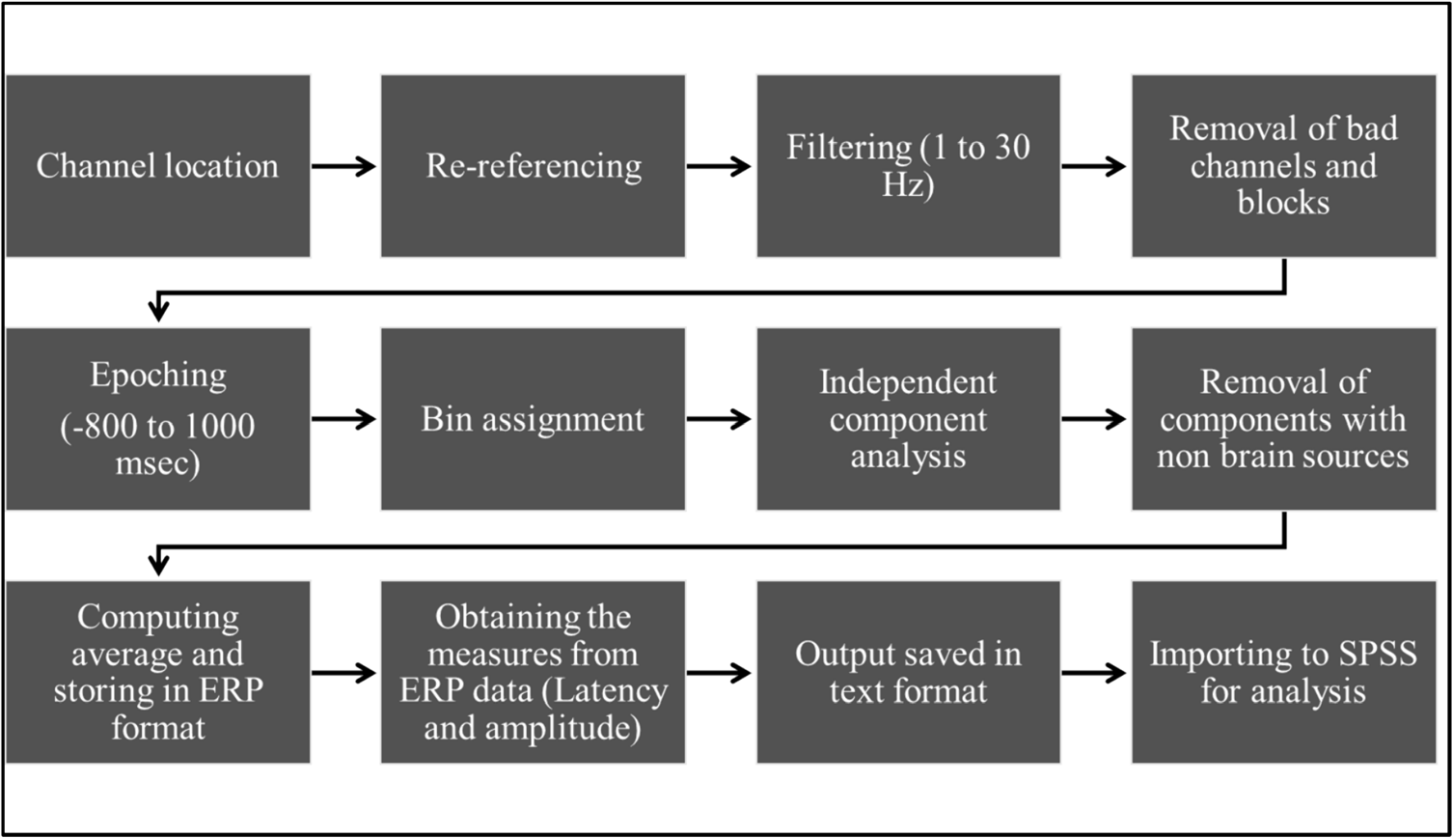
Standard preprocessing pipeline for EEG data analysis

The EEG data were preprocessed using EEGLAB v2021.0 (Delorme and Makeig 2004) running on Matlab R2018b. EEGLAB is a freely available academic software used to process electrophysiological data. EEG lab helps to process high-density EEG and other brain data through its interactive graphic user interface. The standard preprocessing pipeline (shown in Fig. 3) was followed for this study. Based on visual inspection, bad blocks were marked for exclusion from further analysis. Channels were re-referencing to average reference, and the recording reference channel (Cz) was included in the analysis. EEG signals were bandpass filtered between 1 and 30 Hz and then epoched between − 800 and 1000 msec in ERPLAB version 8.10 (Lopez-Calderon and Luck 2014).

Further, the EEG data were decomposed using Independent Component Analysis (ICA) to separate the nonbrain activities. After which, each decomposed component was classified using an automatic independent component classifier called “ICLables.” Based on the labeling done by ICLables, components like blinks, line noise, muscle artifacts, EKG, and other nonbrain activities were marked for rejection and then removed manually. Following this, ERP was computed by averaging across the trials and loaded to a measurement tool in ERPLAB to quantify N100 and P300 components. The P300 component is a positive peak following N2 in the latency range of 300–600ms. N100 is the first negative component after stimulus onset in the 80−120 ms latency range. The mean amplitude is the average amplitude in microvolt in the defined latency range. Peak Latency is the latency of amplitude maxima in the defined latency range.

#### Data extracion

Behavioral responses were evaluated based on mean reaction time and accuracy and derived network scores representing differences between conditions. Specifically, the alerting network score was calculated as the reaction time difference between double cue and no cue conditions, the orienting network score as the reaction time difference between spatial cue and center cue conditions, and the executive control network score as the reaction time difference between incongruent and congruent conditions. As per the literature, the main aim of ANT is to infer the triple networks of attention as per the Posner model of the attention network. Hence, the current study mainly highlights the network function, while overall statistics is given for the raw scores.

Further, the electrophysiological equivalent of attention networks (Ma et al. 2023; Neuhaus et al. 2010) is calculated as alerting network = target locked N100 in no cue versus double cue condition; orienting network = target locked N100 in the center versus spatial cue condition finally, executive network = overall P300 amplitude in congruent versus incongruent condition. In the current study, the parietal electrodes P3 and P4 were used to measure alerting and orienting attention, while the electrode Pz was used to measure executive control attention. These electrodes are known to show significant changes in early attention and executive function, as reported by Luck (2012), Mangun & Hillyard (1988), Ma et al. (2023), and Neuhaus et al. (2010). Following the stimulus presentation, the study analyzed the average amplitude of N100 and P300 at 150–250ms and 300–600ms, respectively (Ma et al. 2023).

#### Statistical analysis

All data was analysed using IBM SPSS Statistics 22. The normality of the data was checked using the Shapiro-Wilk test. As the data was found to be normally distributed, the following statistical tests were carried out: An independent t-test was used to compare the groups in each attentional network score. For electrophysiological measures, a 2 × 2 × 2 repeated measures ANOVA was used to analyze alerting and orienting networks, and a 2 × 2 repeated measures ANOVA was used to analyze the executive control network. All post-hoc analyses were conducted, applying Bonferroni corrections to account for multiple comparisons. A Pearson product correlation coefficient was used to correlate PTA, SNR loss, and attentional network scores.

## Results

### Behavioral measures

The raw scores, which consist of cue-wise mean reaction time across the flankers and their standard deviations, are displayed in Table 3.

**Table 3:** Cue-based mean reaction time with standard deviation across the flankers

Flankers	Group	Cues (reaction time in milliseconds)			
		No cue	Centre cue	Double cue	Spatial cue
Neutral	Hearing Loss	759.41 ± 97.63	729.58 ± 91.22	713.92 ± 104.74	719.75 ± 123.91
	Normal hearing	633.67 ± 51.18	613.82 ± 58.63	559.09 ± 130.87	594.89 ± 80.59
Congruent	Hearing Loss	789.22 ± 100.69	772.77 ± 107.25	731.14 ± 87.21	718.24 ± 91.20
	Normal hearing	660.88 ± 60.88	624.59 ± 59.18	597.89 ± 67.95	589. 84 ± 61. 93
Incongruent	Hearing Loss	845.20 ± 115.99	842.83 ± 125.86	864.31 ± 138.55	830.19 ± 137.07
	Normal hearing	694.21 ± 77.50	693.39 ± 67.17	689.56 ± 60.46	673.94 ± 63.61

#### Attentional networks/index

**Fig. 4:**
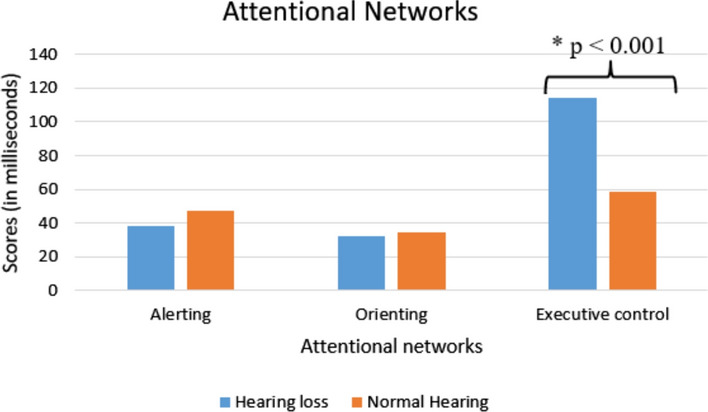
The means scores of attentional networks of both the groups

Attentional network scores in milliseconds are depicted in Fig. 4. Independent t-test results revealed a significant difference between the hearing-impaired group and the normal hearing group in executive control network scores (t (30.150) = 5.006, *p* < 0.001). Whereas alerting (t (41) = 0.746, *p* = 0.460) and orienting networks scores (t (41) = − 0.420, *p* = 0.677) did not show a statistically significant difference.

In terms of accuracy, the results revealed no significant difference between the groups across all three attention networks; alerting (t(24.906)= − 1.433, *p* = 0.164), orienting (t(27.390) = − 1.457, *p* = 0.157), and executive scores (t(26.108) = -1.544, *p* = 0.135).

### Electrophysiological measures

#### Alerting network

**Fig. 5:**
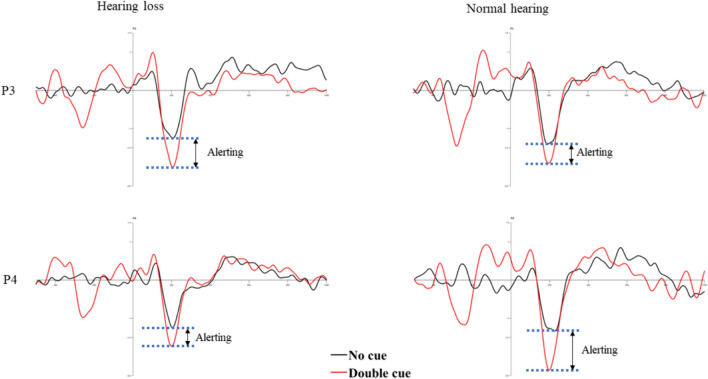
Illustration of alerting effect in both groups

**Fig. 6:**
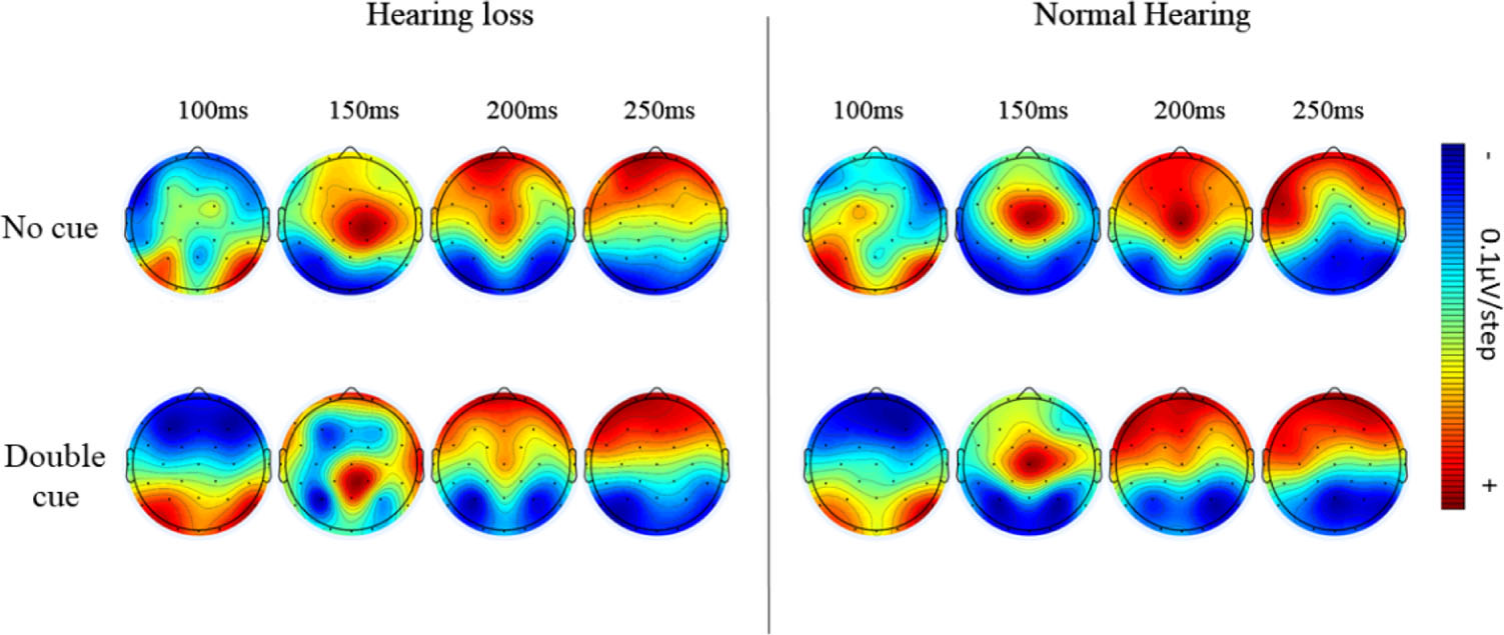
The mean current density maps of the alerting network. The maps represent a two-dimensional top view within the time frame of 100−250 ms after the onset of both the cue and target (target N100). The top row of the maps shows the one with no cue, while the bottom row displays the map with a double cue

Target locked N100 of no cue and double cue condition was analyzed to explore the alerting network using repeated measures ANOVA. The results showed significant main effects of group (F (1, 655.437) = 6.098, *p* = 0.018, η^2^ = 0.129), revealing larger N100 amplitude in the normal hearing group compared to the individuals with hearing loss in both no cue and double cue conditions (*p* = 0.018). Similarly, a significant main effect of the cue condition (F (1, 505.083) = 40.451, *p* < 0.001, η^2^ = 0.497) showed a larger N100 amplitude in the double cue condition than the no cue condition in both groups (*p* < 0.001). No significant interaction was reported between the group, cue conditions, and channels (ps > 0.05). Target-locked grand averages ERP waveforms and corresponding topographic distribution depicting the alerting effect are shown in Figs. 5 and 6.

#### Orienting network

Further, target-locked N100 of center cue and spatial cue condition was analyzed to investigate the orienting effect using 2 groups × 2 conditions × 2 channels ANOVA.

**Fig. 7:**
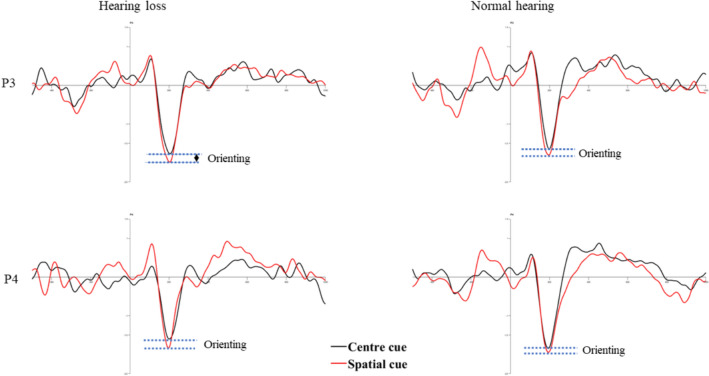
Illustration of orienting effect in both groups

**Fig. 8:**
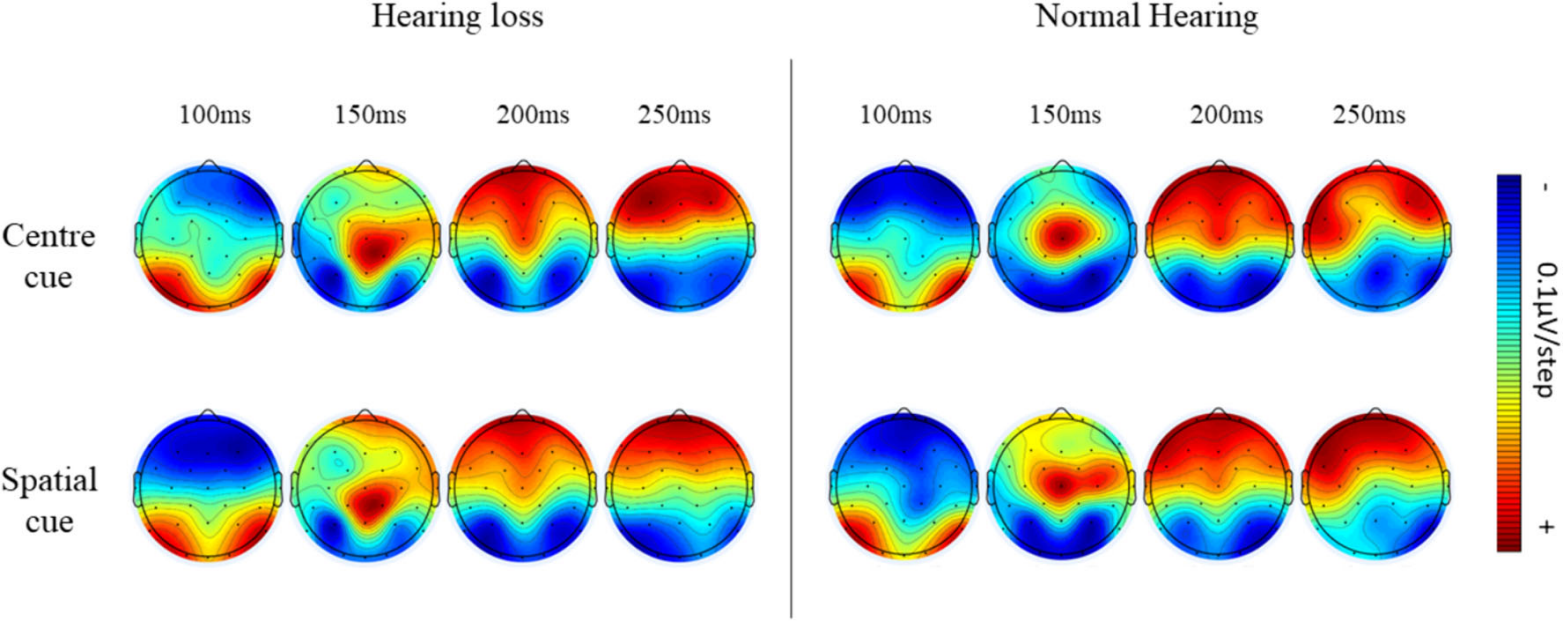
The mean current density maps of the orienting network. The top row shows the map with a center cue, while the bottom row shows the map with a spatial cue. These maps represent a two-dimensional top view within the time frame of 100 to 250ms after the onset of both the cue and target (target N100)

The results revealed a significant main effect of cue condition (F (1, 38.142) = 5.237, *p* = 0.027, η^2^ = 0.113) reflecting larger N100 amplitude in spatial cue condition than center cue condition in both the groups (*p* < 0.001). The significant main effect of group (F (1, 645.364) = 6.289, *p* = 0.016, η2 = 0.133) revealed larger N1 amplitude in normal hearing individuals than those with hearing loss (*p* = 0.014). There was no significant interaction between the group, cue, and channels (F (1, 55.002) = 7.551, *p* = 0.009, η^2^ = 0.156). Target-locked grand averages ERP waveforms and corresponding topographic distribution depicting orienting effect are shown in Figs. 7 and 8.

#### Executive control network

**Fig. 9:**
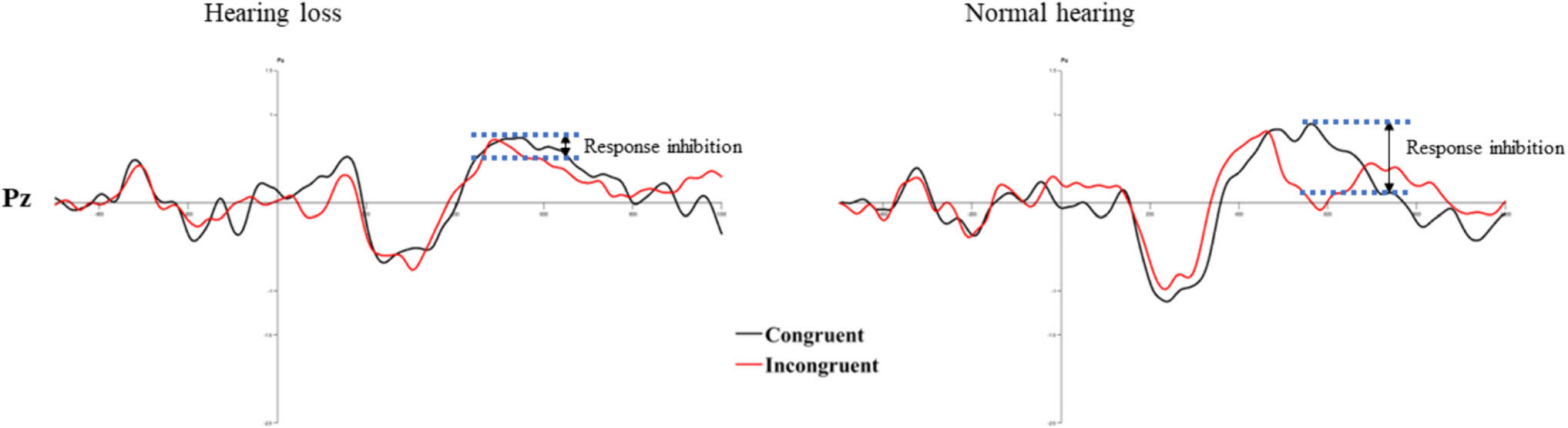
Illustration of response inhibition in both groups

**Fig. 10:**
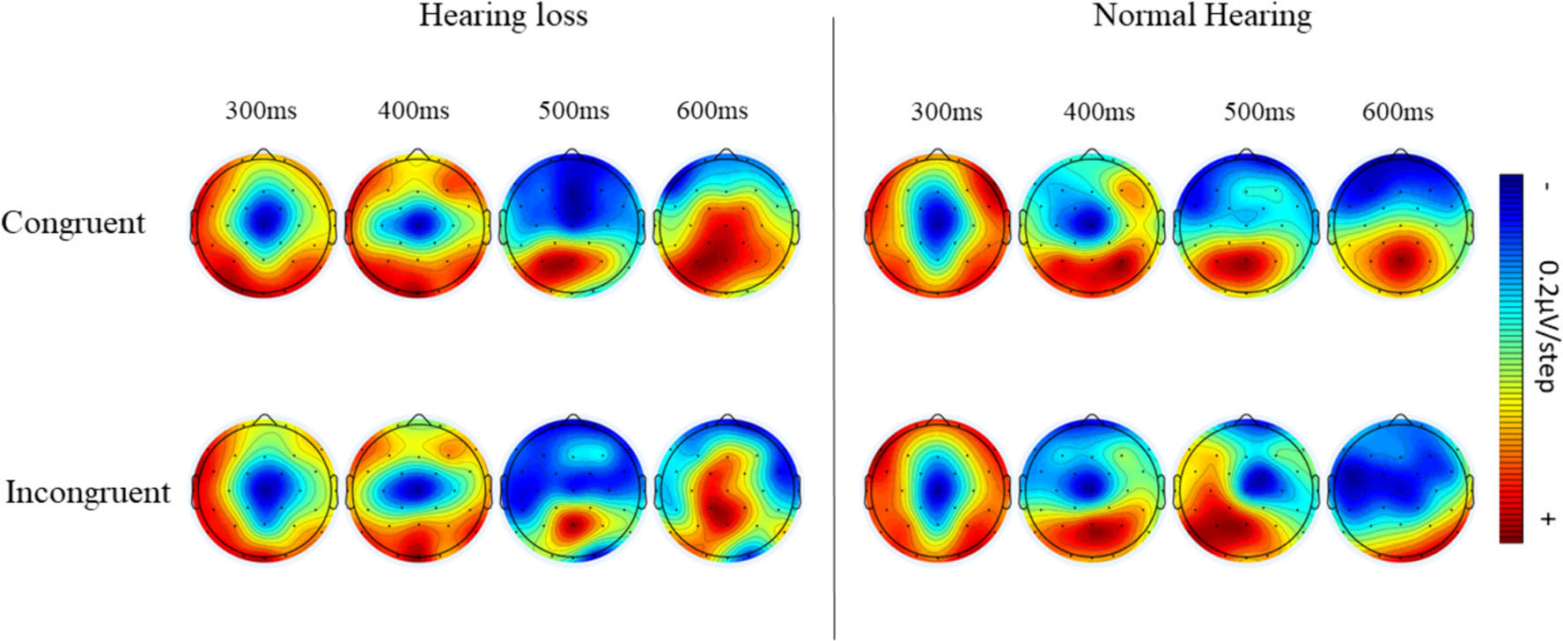
The mean current density maps of the executive control network. The top row displays the map with congruent conditions, while the bottom row shows the map with incongruent conditions. The maps represent a two-dimensional top view within 300–600 ms after the onset of the target (target P300)

P300 amplitude in congruent and incongruent conditions was used to explore the executive control network. The two levels of group × two levels of flanker conditions ANOVA resulted in a significant main effect of flanker condition (F (1, 68.436) = 6.296, *p* = 0.016, η^2^ = 0.136) revealing reduced amplitude for the incongruent condition than the congruent condition (*p* = 0.034). Similarly, a significant effect of group (F(1, 615.158) = 5.464, *p* = 0.025, η^2^ = 0.120) reflected in larger P300 amplitude in individuals with normal hearing sensitivity compared to individuals with hearing impairment (*p* < 0.001). Figure 9 illustrates the response inhibition in the P300 range in grand averaged ERP waveforms, and Fig. 10 shows the difference in topographic distribution in the corresponding latency range.

### Correlation analyses

Table 4 shows the correlation between SNR loss, PTA and behavioural attentional networks scores using Pearson’s correlation. The results revealed significant positive correlation between executive scores and PTA as well as SNR loss (*p* < 0.001). Similarly, electrophysiological results, SNR loss and PTA revealed negative correlation with orienting attention network scores.

**Table 4:** Correlation between SNR Loss, PTA and attention network scores

	PTA	Electrophysiological scores			Behavioural scores		
		Alerting	Orienting	Execution	Alerting	Orienting	Execution
SNR Loss	0.863**	− 0.163	-0.374*	− 0.135	-0.039	− 0.062	0.516**
PTA	1	− 0.123	-0.375*	− 0.031	0.067	− 0.183	0.518**

*Significant at the level of 0.005

**Significant at the level of 0.001

### Post hoc power analysis and effect size

A post hoc power analysis was conducted to determine whether the study could detect a significant effect at α = 0.05. The analysis showed that with a sample size of 40 participants, the power achieved for detecting a significant effect was 0.99 and 0.997 for electrophysiological and behavioral measures, respectively. Achieving power values close to 1.0 means that the study is highly likely to detect a true effect if it exists. Therefore, with the achieved power values of 0.99 and 0.997, this study is highly powered and can detect a significant effect when it is present. Further, Cohen’s d was used to calculate the effect size for each attention network to quantify the difference between groups. The estimates are presented in Table 5. The executive attention network showed the largest effect size for behavioral and electrophysical measures, suggesting consistent and robust differences between the groups. On the other hand, Alerting and orienting networking showed a larger effect size for electrophysiological measures than behavioral measures.

**Table 5:** Estimates of the effect size for attention networks

	Alerting attention	Orienting attention	Executive attention
Behavioral measures	0.22	0.13	1.49
Electrophysiological measures	0.43	0.84	1.25

## Discussion

The results of the present study give compelling evidence for the negative impact of age-related hearing loss on attention networks, consisting of both behavioral and electrophysiological measures.

The results revealed longer reaction times and poorer executive scores in older individuals with hearing loss. This result suggests the impact of hearing loss on the high-level cognitive process involved in cognitive flexibility and attentional control. Earlier studies using tests like Stroop reported impaired executive function in individuals with hearing loss despite age (Lin et al. 2011; Valentijn et al. 2005). However, studies exploring attention deficits in the framework of ANT among healthy older individuals are sparse. Williams et al. (2016) showed impaired executive attention networks in healthy older individuals. The current study is the first attempt to explore the attention network framework among older individuals with hearing impairment.

The findings of our study suggest that these individuals with hearing loss exhibit even more impaired executive function than their normal-hearing counterparts. Some studies question the construct of visual-cognitive tests in hearing-impaired individuals (Shen et al. 2020) but the auditory cognitive test results in consistent overestimation of cognitive deficit (De Silva et al. 2008; Dupuis et al. 2015). Assessing cognition in the auditory modality in the hearing impaired could be influenced by an acute increase in the cognitive load specific to features like the stimulus’s intensity and familiarity. ANT is a well-established test showing a cognitive influence in many clinical populations. In the current study, impaired executive attention assessed in visual modality among hearing-impaired older individuals suggests long-term effects of cognitive resource allocation deficit. Also, it is noteworthy that the current study included individuals only with mild and moderate hearing impairment. This suggests that even a lesser degree of hearing loss would negatively impact executive attention function.

The current study shows that a higher degree of hearing loss results in poorer executive function. The same relationship exists for central auditory processing tests like SPIN. Similar results support earlier findings using other executive function tests (Lin et al. 2011). Consideration of SNR loss in our study additionally gives more insight into the functional deficit of an individual with hearing loss.

In the current study, alerting and orienting scores between older individuals with hearing loss and normal hearing are similar. These findings are exploratory as there are no previous studies to compare. However, studies show preserved alerting and orienting attention in healthy older individuals and hearing-impaired younger adults (Ma et al. 2023; Williams et al. 2016). These findings are discussed later in this section, along with ERP findings.

The current study also evaluates the neurophysiological responses to the ANT using simultaneous EEG recording. It aids in elucidating the different stages of stimulus processing at the cortical level (Polich 2007). ERP results suggest significant differences in all three attention networks in older individuals with hearing loss. The P300 amplitude was significantly smaller than the controls across all conditions, irrespective of congruency and cue types. The electrophysiological index of the executive attention network is seen as a suppression of P300 amplitude in incongruent trials (Coles et al. 1985; Pfefferbaum et al. 1985). This is seen in controls but not in older individuals with hearing loss. Such lack of inhibition is postulated due to a deficit in resource allocation in conditions like incongruent flankers (Neuhaus et al. 2010). This ascertains the observed behavioral deficit, wherein individuals with hearing loss allocated fewer attentional resources to conflict resolution tasks. Thus, reflecting the negative influence of hearing loss on higher-level attentional functions involved in inhibiting irrelevant stimuli and conflict resolution.

In addition, there was a significant difference in alerting and orienting attention, which is evident in electrophysiological response but not in reaction time or accuracy. The N100 amplitude difference between no cue and double cue condition is considered an index of alerting attention, and the difference between spatial cue and centre cue condition is as orienting attention (Neuhaus et al. 2010). In the current study, overall N100 amplitudes were smaller in older individuals with hearing loss than their normal counterparts. The result reflects the influence of altered early attention processes in older individuals with hearing loss.

Previous literature shows that younger deaf individuals generally exhibit alerting attention network deficits while preserving orienting attention (Ma et al. 2023). On the other hand, healthy older individuals showed intact alerting and orienting networks (Williams et al. 2016). However, our study focused on older individuals with hearing loss revealed both alerting and orienting attention impairments. These findings highlight the specific impact of hearing loss on the initial stages of attentional processing.

On comparison of the ANT behavioral scores and the concurrent ERP measures, it is clear that the inferences were consistent with executive attention but not for orienting and alerting attention. ERPs showed impaired orienting and alerting attention, but the reaction times were not different. It could be possibly due to the difference in the measures’ sensitivity. ERPs are covert responses sensitive to the time course of cortical activation of sensory and cognitive processing stages. However, the behavioral are overt responses that reflect the end stage of the above processes (Alderman et al. 2019). The disagreement between behavioral reaction time and ERPs can be attributed to the subtle attention dysfunctions in alerting and orienting networks. It indicates that the subtle alterations in alerting and orienting attention networks would not be reflected in behavioral responses. At the same time, covert ERP measures could detect even subtle changes in the attention networks. Thus, the current study infers that individuals with hearing loss might have subtle deficits in alerting and orienting attention.

Additionally, the results from the post hoc power analysis indicate that the study was well-equipped to detect significant effects, with power values of 0.99 and 0.997 for electrophysiological and behavioural measures, respectively. Such power values, being close to 1.0, suggest a high likelihood of the study detecting true effects if they exist. This high level of power is crucial for ensuring the reliability of the study’s findings, as it minimizes the risk of Type II errors, where a true effect is present but goes undetected.

Moreover, Cohen’s d was utilized to calculate effect sizes for each attention network, revealing significant differences between groups. The executive attention network showed the largest effect size for both behavioural and electrophysiological measures. This suggests that the differences between groups were most pronounced in tasks involving the executive attention network, indicating robust and consistent disparities. In contrast, the alerting and orienting networks exhibited larger effect sizes in electrophysiological measures compared to behavioral measures. This disparity points to the possibility that electrophysiological measures may be more sensitive or specific in detecting differences in these networks compared to behavioral measures.The findings underscore the importance of choosing appropriate measures and analyses to capture and interpret the nuances of attention networks accurately. The high power of the study ensures confidence in the detected effects, while the effect size calculations provide valuable insights into the magnitude and significance of the differences observed.

Overall, study provides compelling evidence of impaired executive attention in older individuals with hearing loss. In addition, subtle dysfunction in alerting and orienting networks is also reported in older individuals with hearing loss. This suggest a complex link between age-related hearing loss and deficits in the attentional network. Individuals with hearing loss have to put forth more effort to understand the given auditory signal, which means that they end up using cognitive resources that are also needed for other tasks (Chern & Golub, 2019; Huang & Lin, 2024; Lin et al., 2013; Nixon et al., 2019; Ray, Popli, & Fell, 2018; Uchida et al., 2019; Zhao et al., 2024). This reallocation of cognitive resources to compensate for the degraded auditory input in the long term leads to attention deficits, even in nonauditory tasks (Pichora-Fuller 2006, 2016; Pichora-Fuller et al. 2016). Campbell and Sharma (2013) revealed that adults with early-stage hearing loss showed reduced activation in auditory regions and increased activation in frontal and prefrontal cortices. These areas are linked to working memory and executive functions, indicating greater cognitive load and listening effort (Harris et al. 2009; Lin et al. 2014; Peelle et al. 2011; Wingfield et al. 2006). This explains the significant correlation between the speech-in-noise scores and executive attention in the current study. Additionally, our study highlights the crucial role of hearing sensitivity measures in executive function impairments in older individuals. The study also suggests that hearing loss could independently lead to attention deficits in older individuals despite their age and education. Further investigation of alerting and orienting attention networks across the severity and duration of hearing loss is recommended.

## Implications

These findings underscore the complex interplay between hearing loss and attentional network deficits, emphasizing the need for early detection and intervention strategies to address the multifaceted challenges faced by individuals with hearing loss. The study also emphasized the importance of integrating cognitive assessment as a standard component of evaluations for older individuals with hearing impairments. The study revealed that hearing loss can potentially impact cognitive abilities such as attention, which may, in turn, affect the effectiveness of hearing aid interventions. As a result, further investigations are warranted to gain a deeper understanding of how these interconnected factors influence the overall outcomes of hearing aid usage.

## Limitations

The main aim of this study is to investigate how hearing loss affects the older individuals’ attentional ability. Since there is a known disparity in performance between younger and healthy older adults when using the ANT, a younger control group was not included in this study for comparison. Therefore, the results need to be evaluated in conjunction with research on healthy younger adults. Both groups consist of 20 participants, and the power analysis has verified that the sample size is adequate for interpretation.

## Data Availability

Data will be made available upon request. The ANT paradigm used in the study has been uploaded to OSF (OSF Link: https://osf.io/jmn8s/ ).

## Abbreviations

ANT: Attention network test
ERPs: Event related potentials
PTA: Pure tone average
SNR loss: Signal to noise ratio loss
RMANOVA: Repeated measures analysis of variance
RT: Reaction time
